# A method for calculating adherence to polypharmacy from dispensing data records

**DOI:** 10.1007/s11096-013-9891-8

**Published:** 2013-11-29

**Authors:** Isabelle Arnet, Ivo Abraham, Markus Messerli, Kurt E. Hersberger

**Affiliations:** 1 Pharmaceutical Care Research Group, Department of Pharmaceutical Sciences, University of Basel, Klingelbergstr. 50, 4056 Basel, Switzerland; 2 Center for Health Outcome and Pharmacoeconomic Research, University of Arizona, 1295 N. Martin, Tucson, AZ 85721 USA

**Keywords:** Adherence, Compliance, Daily polypharmacy possession ratio, Medication possession ratio, Pharmacy claims, Polypharmacy, Refill data

## Abstract

*Background* Several measures for calculating adherence to one medication from dispensing data records have been proposed, but the nomenclature is inconsistent and computations vary. The same measures, like the medication possession ratio (MPR), have been used for multiple medication regimens, and have tended to over- or under-estimate adherence rates. *Objective* To demonstrate the impact of varying elements in MPR to a single medication regimen; to define standards for the estimation of adherence to polypharmacy; to propose a new method for calculating adherence to polypharmacy; to face validate it. *Setting* Face validity of the proposed method. *Method* Variations in the MPR formula were simulated. Standards for the estimation of adherence to polypharmacy were defined. A new method to calculate adherence to polypharmacy was established. Its face validity with three illustrative cases obtained from a pharmacy refill database was assessed. *Main outcome measure* Adherence rate to polypharmacy from refill data records. *Results* MPR to a single medication is operationalized in the numerator and denominator and is influenced by the parameters like observation period, medication gaps, overlap. For polypharmacy, an average MPR is commonly used, which is not accounting for the specificity of multiple medications, and hence overestimating adherence rate. We propose the daily polypharmacy possession ratio (DPPR) as an index of adherence to polypharmacy. It estimates the proportion of time a patient had medication available for use by considering the presence or absence of multiple medications on each day in the observation period. We calculated possession rates from refill histories over 31 months (January 1, 2011–July 31, 2013) for three illustrative patients. The average MPR estimates were 80 % for a patient with 6 medications/20 refill dates, 90 % for a patient with 4 medications/11 refill dates, and 89 % for a patient with 3 medications/17 refill dates. The corresponding DPPRs were 75, 88 and 99 %, indicating overestimations by 5 and 2 %, and underestimation by 10 %, respectively. *Conclusion* The DPPR accounts for the specificity of polypharmacy including number of medications, medication switching, duplication, overlapping. Research is needed to further confirm the validity of this new index.

## Impact of findings on practice

- The Daily Patient Possession Ratio (DPPR) offers clinicians and researchers a method for estimating adherence to polypharmacy regimens.
- When calculating adherence to polypharmacy, the DPPR avoids the overestimation inherent to using single-medication records.

## Introduction

Because a patient’s medication-taking behaviour is a prerequisite for evaluating the effectiveness of medications [1] accurate and consistent measurement of adherence is critical. The advance of computerized pharmacy records in developed countries that use medical informatics in their health system enables the assessment of adherence to an index medication based on refill patterns [2]. Several measures for calculating adherence rate from secondary database have been proposed, such as: medication possession ratio (MPR) and related measures of availability; discontinuation/continuation; switching; medication gaps; refill compliance, and retentiveness/turbulence [3]. All have in common that they measure the timeliness of prescription or refills, not actual drug-taking, and use the medication exposure time to estimate adherence. Consequently, the measures quantify the patient’s possession of medication and, thus, calculate the highest possible level of medication consumption over a particular time frame. Although there is no gold standard, MPR is the most commonly used measure. It is calculated by dividing the days’ supply of a medication dispensed by the number of days in the time interval of interest. Another often used measure is the proportion of days covered (PDC), which represents the proportion of days a patient has a medication available in a given period of time and uses indices truncated at 1.0 [4]. These measures are widely used because dispensing databases contain the necessary elements for calculation: (a) the *quantity dispensed*, which usually is the package size or a multiple of it dispensed at one time; or alternately for dispensing from bulk stock the number of medications supplied at one time; (b) the *prescribed daily dosage*, or the amount of medication to be consumed per day, which is calculated as (pills per dose) × (dose per day); and (c) the *number of days’ supply*, that is, the quantity dispensed divided by the prescribed daily dosage.

Being derived from longitudinal dispensing databases, the MPR and PDC can quantify long-term adherence and associated outcomes. However, five definitions influence the calculation of these measures and explain the variations in results often seen. First, the *observation period*, i.e., the length of the time over which adherence is assessed, may start and end at a specific fill and refill date; on arbitrary start/stop dates that are set as the index or inventory date and are independent from fills and refills; or a combination of a fixed and an arbitrary date. Second, an *initial/terminal gap* between dates of first/last fill and arbitrary start/end dates may be present and can be quantified as a proportion of time without supply. Third, an *interim gap* may exist between refills when prior supply is depleted before refill supply is available. Fourth, the *number of days’ supply* dispensed at any fill/refill event may vary and requires adjustments in the calculations. Alternately, and fifth, *overlap* may occur as refill precedes depletion of the quantity from a prior dispensing, and leads to stock piling of accumulated supply. These five sources of bias may lead to an under- or over-estimation of adherence to a single-medication.

The effects of these five sources of bias are likely to be amplified when adherence to a polypharmacy regimen is estimated using methods for single-medication adherence such as the MPR or, as commonly done, averaging the MPR of each medication in the polypharmacy regimen. Polypharmacy is common due to comorbidities [5], an aging population [6], clinical practice relying on multi-drug combinations [7], or evidence-based guidelines recommending synergistic drug combinations [8]. Polypharmacy is different from regimen complexity, which refers to the number of daily doses for a medication, the presence of non-oral routes of administration, and the need for specific dosing instructions [9]. Because polypharmacy is known to be associated with medication non-adherence [10] because of the greater number of medications that can be missed on a daily basis, the assessment of adherence to the entire polypharmacy regimen is essential. Further, because irregular and inconsistent intake of one or more drugs in a polypharmacy regimen is common and may impact on clinical outcomes, assessment of adherence to polypharmacy is clinically relevant. The few studies that have attempted to calculate adherence to several concurrent medications have averaged the indices obtained for each of the single-medications [11–15]. This method has been shown to overestimate [16] but may also underestimate adherence to polypharmacy regimens.

## Aim and objectives

In the absence of an integrated method for assessing adherence to polypharmacy regimens and the estimation errors likely from averaging methods, our aim was to develop a new method for quantifying adherence to polypharmacy regimens. Five objectives applied: (1) to document the estimation bias in single-medication adherence as a function of the sources of variation identified above; (2) to document the estimation bias resulting from averaging single-medication methods to polypharmacy regimens; (3) to specify the standards for calculating an integrated measure of polypharmacy adherence; (4) to define the proposed method for calculating adherence to polypharmacy regimens; and (5) to establish initial face validity of the method by applying to three illustrative cases obtained from the dispensing records of a community pharmacy.

## Methods

### Estimation bias in single-medication adherence

We constructed a hypothetical refill scenario commonly seen in reimbursement records for medicines for long-term conditions in order to illustrate the impact of variable elements on the calculations of adherence to single-medication such as hypertension. We selected seven dispensations in analogy to Steiner et al. [14] and calculated several indices. Two different observation periods of 250 days each were used: (1) from the first fill to the last refill date; and (2) over two arbitrary dates. Four calculations were performed:

1. The *proportion of supply between dispensations* or *adherence in one refill interval* (*A*_*n*_ */B*_*n*_), calculated as the days’ supply obtained at the beginning of a specific interval divided by the days elapsed before the subsequent fill and expressed as a percentage.
2. The *days without supply between dispensations* or *gaps in one refill interval* (*G*_*n*_ = *B*_*n*_ − *A*_*n*_), calculated as the days elapsed before the subsequent fill i.e., the number of days between dispensations, minus the days’ supply obtained at the beginning of the interval.
3. The *proportion of time with adequate supply* or *medication possession ratio* (*∑A*_*n*_/*∑B*_*n*_), calculated as the total days’ supply obtained over the observation period and across all time intervals divided by the number of days of the observation period and expressed as a percentage.
4. The *proportion of time without adequate supply* or *gaps over all refill intervalls* (*∑G*_*n*_/*∑B*_*n*_), calculated as the total days of gaps (+) or surplus (−) divided by the total days to next dispensation or to end of observation period; that is, the cumulative sum of the number of days between dispensations minus the total days’ supply divided by the number of days in the observation period.

### Estimation bias in polypharmacy adherence calculated with averaging methods

We again constructed a hypothetical scenario, this one involving 3 medications with a combined 15 refills [17] and an observation period beginning with the initial fill at the start date and ending with the medication review. Medication 1 came in a package size of 14 with seven refills at days 1, 15, 29, 43, 57, 71, 101 and end date at 110 days. Medication 2 came in a package size of 30 with four refills at days 1, 41, 61, 120 and end date at 120 days. Medication 3 came in package size of 60 with four refills at days 1, 31, 51, 101 and end date at 110 days.

### Specification of standards

On the basis of the above bias estimation exercises, prior review work, and literature evidence, calculation standards were set to assure uniformity in calculations.

### Development of method

A proposed method based on these standards was developed and evaluated for arithmetic accuracy.

### Initial assessment of face validity

We applied the method to three illustrative cases varying in the number of medications and refills obtained from the dispensing data records of a community pharmacy in Basel, Switzerland.

## Results

### Estimation bias in single-medication adherence

**Fig. 1:**
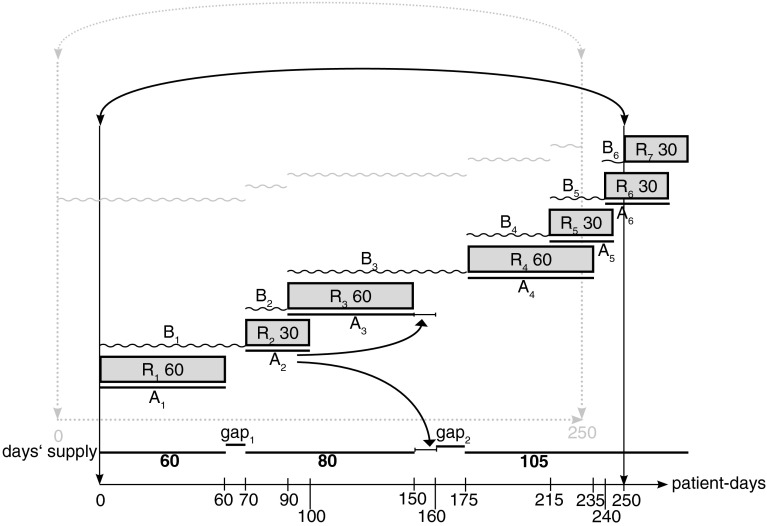
Scenario of adherence to a single-medication, starting at the first fill (*dark*, *bold line*) or an arbitrary date (*grey*, *dotted lines*) over an observation period of 250 days (arbitrary end date). R_n_X = refill number and quantity dispensed; A_n_ = number of days’ supply; B_n_ = interval between dispensations; gaps indicate number of days with no medication. *Note* the *arrows* from R_2_ to R_3_ indicate carryover of excess medication from one interval to the next interval

Figure 1 depicts the adherence calculations for a patient with a chronic condition with a hypothetical refill scenario of a medication to be taken once daily with 7 dispensations in analogy to Steiner et al. [14]. Table 1 summarizes the calculated adherence rates between each refill event and the next.

**Table 1:** Estimates of single-medication adherence between each refill event and the next, for each observation period (starting at a refill date or an arbitrary date)

Refill event	R1	R2	R3	R4	R5	R6
Start at refill date	60/70 (86 %)	30/20 (150 %)	60/85 (71 %)	60/40 (150 %)	30/25 (120 %)	30/10 (300 %)
Start at arbitrary date	60/90 (67 %)	30/20 (150 %)	60/85 (71 %)	60/40 (150 %)	30/15 (200 %)	–

R(number) refers to interval starting at a given refill event and ending at the next refill event; e.g., R1 is refill event 1 and the interval ends with R2

Table 2 presents the days without supply between dispensations (or gaps).

**Table 2:** Days without supply between dispensations (or gaps), for each observation period (starting at a refill date or an arbitrary date)

Refill event	R1	R2	R3	R4	R5	R6
Start at refill date	70–60 (10)	20–30 (−10)	85–60 (25)	40–60 (−20)	25–30 (−5)	10–30 (−20)
And with carry over of oversupply	70–60 (10)	20–20 (0)	85–70 (15)	40–60 (−20)	25–30 (−5)	10–30 (−20)
Start at arbitrary date	90–60 (30)	20–30 (−10)	85–60 (25)	40–60 (−20)	15–30 (−15)	–

A positive value indicates a lack of supply, a negative value indicates a surplus of supply

Table 3 summarizes the MPR results. The overall possession rates are 108 %/84 % if calculations consider all values without the last refill, and 93 %/87 % if single values are capped at 1.0, underscoring that adherence is underestimated with truncated values.

**Table 3:** Medication possession ratio (MPR) calculated over all refill intervalls, with or without the last refill, and with single values capped at 1.0, for each observation period (starting at a refill date or an arbitrary date)

Specifications	With last refill	Without last refill	With single values capped at 1.0
Start at refill date	300/250 (120 %)	270/250 (108 %)	(0.86 + 1 + 0.70 + 1 + 1 + 1)/6 (93 %)
Start at arbitrary date	240/250 (96 %)	210/250 (84 %)	(0.67 + 1 + 0.70 + 1 + 1)/5 (87 %)

Table 4 presents the proportion of time without adequate supply (or gaps) over all refill intervals. The proportion of gaps is −0.08/0.04 if calculations consider all values and 0.14/0.22 if negative values are set to zero, thus masking the surplus (negative gaps’ value). If accumulated oversupply is assumed to be used when the supply is exhausted and carryover from one interval to the next is allowed, the proportion of time without medication declines from 35 to 25 days, which corresponds to an overall 4 % improvement in supply.

**Table 4:** Proportion of time without adequate supply (or gaps) over all refill intervals, with all values or negative values set at zero (no surplus), for each observation period (starting at a refill date or an arbitrary date)

Specifications	With all values	With negative values set at 0 (no surplus)
Start at refill date	(10 − 10 + 25 − 20 − 5 − 20)/250 (−0.08)	(10 + 0 + 25)/250 (0.14)
Start at arbitrary date	(30 − 10 + 25 − 20 − 15)/250 (0.04)	(30 + 0 + 25)/250 (0.22)

### Estimation bias in polypharmacy adherence calculated with averaging methods

**Fig. 2:**
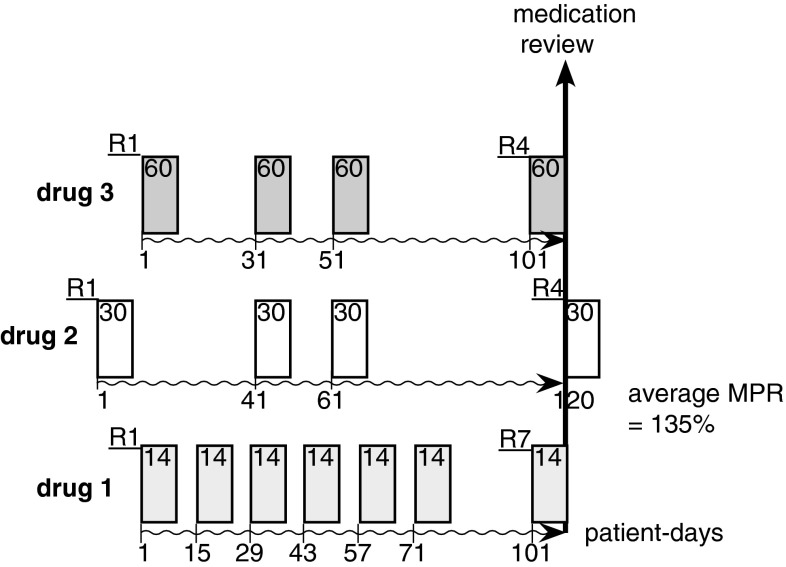
Scenario of adherence to a combined drug regimen for a patient (with a chronic condition) with a medication to be taken once daily in analogy to [17]. The refills of 3 medications are depicted with 15 dispensations over an observation period defined between the first fill (R1 at day 1, start date) and a medication review (arbitrary end date). *R* refill number; *box with number* quantity dispensed

Figure 2 graphs the average MPR calculation with a hypothetical scenario of 3 medications with a combined 15 refills. Note that the observation period begins with the first refill at start date “day 1” and runs until the medication review (an arbitrary date). The MPR for medication 1 is (7 × 14)/110 = 89 %; for medication 2 it is (4 × 30)/120 = 100 %; and for medication 3 it is (4 × 60)/110 = 218 %. Hence the average MPR is [(7 × 14) + (4 × 30) + (4 × 60)]/(110 + 120 + 110) = 135 %, denoting an overconsumption of medication.

### Specification of standards

The average MPR calculation does not control for the influence of several medications having been prescribed, across varying schedules, and the expectation that patients adhere to all medications regardless of regimen. Further, patients rarely refill a medication on exactly the day following the last day of use of the previous dispensing. In addition, because of different package sizes, patients may have refills due on different dates. As a consequence, they adapt their refill obligation to daily duties and schedules and may refill a prescription earlier (overlap of two dispensations, surplus) or later (gap without supply between two dispensations). Thus, apparently excessive or insufficient refill patterns may be misinterpreted as oversupply or lack of medication, when they may represent daily life conditions such as foresight before holidays or using up all medication before the next refill.

We propose new definitions of the parameters needed to calculate medication possession rates with refill data (Table 5). In the numerator, extra doses beyond the end of the observation period should be excluded (no oversupply); therapeutic switching and therapeutic duplication should be considered as one medication (no duplication), and changes in dosage should be recognised and accounted for. In the denominator, the observation period should start at the first dispensation date, end either at the last refill date or at the medication review date, and cover at least two refills (no gaps). Finally, the carryover of excess medication from one interval to the next interval should be allowed, yet without retroactive compensation.

**Table 5:** New definitions proposed of the parameters required to calculate possession rates with refill data

New definitions of the parameters	Pros
Start the observation period at day 1 with the first dispensation	No artificial initial gaps
End the observation period at the last refill date or at the medication review date	No artificial terminal gaps
Exclude any extra doses of the last dispensation beyond the end of the observation period	No artificial oversupply
Allow the carryover of excess medication from one interval to the next interval, yet without retroactive compensation	No artificial oversupply
Exclude patients with two refills or less	No artificial gaps
Consider therapeutic switching^a^ as one medication and not as a duplication	No artificial duplication
Consider switching from two medications to one combination pill as therapeutic switching, with the first refill in time determining the index medication substituted by the combination pill	No artificial duplication
Allow generic switching and consider as one medication	No artificial duplication
Consider therapeutic duplication^b^ as one medication, with the index medication being the one with the first refill in time	No artificial duplication
Enable changes in dosage according to medical prescription	No artificial oversupply or gap

^a^Medication switch occurs when a subject initially fills a prescription for one product, then at a later point fills a prescription for a different product in the same therapeutic class and never refills the first product within the observation period

^b^Therapeutic duplication is defined as multiple medication use within the same therapeutic class, and can result from therapeutic augmentation; prescription error must be excluded

### New method for calculating adherence to polypharmacy

With these definitions, we posit that the numerator cannot merely be the sum of the days’ supply, that each day should be assessed independently, and that the proportion of daily medications on-hand be calculated. We propose as new index the daily polypharmacy possession ratio (DPPR). The method is as follows: Look at each day in the observation period separately, and determine how many medications are available, set a score between 0 (no medication available) and 1 (all medications available) weighted by the number of medications to be taken each day, resulting in daily scores indicating the proportion of medications available for each day. Sum the scores and divide by the number of days in the observation period to obtain the proportion of all medications available for daily use.

**Fig. 3:**
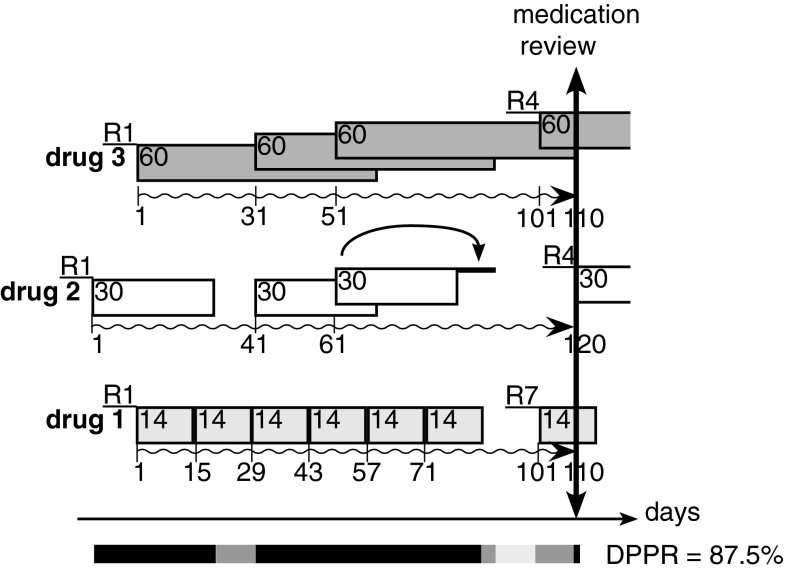
Calculation of daily polypharmacy possession rate DPPR for 3 medications to be taken once daily, with 15 dispensations over an observation period defined between the first fill (R1 at day 1, start date) and a medication review (end date), same as in Fig. 2. Daily possession is depicted as follow: all medications available (score of 1, *black bar*), two medications available (score of 2/3, *dark grey bar*), one medication available (score of 1/3, *light grey bar*). The *arrow* indicates carryover of excess medication from one interval to the next interval

Figure 3 shows the calculation of the DPPR for 3 medications to be taken once daily with 15 dispensations over the same observation period as used in Fig. 2. Each daily score can take a value of 1 (all medications available), 2/3 (two medications available), 1/3 (one medication available) or 0 (no medication available). The sum of the daily scores (10 × 1/1) + (20 × 3/3) + (10 × 2/3) + (54 × 3/3) + (6 × 2/3) + (10 × 1/3) + (9 × 2/3) + (1 × 3/3) is 104.9. Next 104.9/120 = 87.5 % yields the DPPR and represents the weighted percentage of medications available. The accumulated surplus of daily doses is 80 (medication 1). The accumulated number of days with at least one missing dose is 16 for medication 1 (4.7 %) + 30 for medication 2 (8.8 %) = 46 (13.5 %).

The DPPR requires a “supply diary” for each patient-day. Because overuse or excess prescription of medications cannot be detected with the DPPR, the surplus of daily doses and the total number of missed doses (gaps) during the observation period should be evaluated to complete the description of the observed population.

### Initial assessment of face validity

Three patients with polypharmacy using a single community pharmacy in Basel (Switzerland) were selected by the pharmacist who subjectively and clinically identified an adherer, an underadherer and an overadherer. The medication histories between January 1, 2011 and July 31, 2013 were retrieved from the pharmacy database. They include 1 male (M1) and 2 female (F1, F2) patients; aged 72, 78 and 74 years; with 4, 3 and 6 medications daily; and 11, 17, and 20 refill dates over the observation period of 31 months, respectively (Box 1).

**Box 1:** Medication histories of three illustrative patients (M1, F1, and F2) between the start date (January 1, 2011) and an arbitrary review date (July 31, 2013), with indication of the prescribed daily dosage (e.g., 1–0–0 stands for one tablet every morning), number of days in the single intervals and in the observation period (total); quantity dispensed at the refill date and total of days’ supply (calculated as quantity dispensed divided by the prescribed daily dosage), and total number of days without supply (gaps). Asterisks (*) indicate insufficient supply for the next interval

Patient M1	No days in the interval	Quantity dispensed at the refill date of the medication (prescribed daily dosage)			
Refill date		Atorvastatin 20 mg (1–0–0)	Metopropol 25 mg (1–0–0)	Aspirin 100 mg (1–0–0)	Salmeterol 25 μg + fluticason 250 μg (1–0–0)
11.01.11		100	–	90	60*
05.04.11	84	100*	–	196	60*
11.10.11	189	100	100	98	60*
12.01.12	93	100	100	98	60
07.03.12	55	100	100	98	60*
27.06.12	112	0*	0*	0	60
08.08.12	42	100*	100*	98*	120
07.12.12	121	100	100	98	60*
27.02.13	82	100	100	98	60
23.04.13	55	0	0	0	60
14.06.13	52	100	100	98	120
31.07.13	47				
Total	932	900	700	972	780
Total gaps		−96	−23	−18	−238

Calculation: “average MPR” = [(900 + 700 + 972 + 780)/4]/932 = 90 %

DPPR = [(60 × 3/3 + 24 × 2/3) + (60 × 3/3 + 56 × 2/3 + 73 × 1/3) + (60 × 4/4 + 33 × 3/4) + 55 × 4/4 + (65 × 4/4 + 47 × 3/4) + (40 × 4/4 + 2 × 2/4) + (100 × 4/4 + 3 × 2/4 + 18 × 1/4) + (77 × 4/4 + 5 × 3/4) + 55 × 4/4 + 52 × 4/4 + 47 × 4/4]/932 = 88 %

Gaps: mean −10 %

**Table:** 

Patient F1	No days in the interval	Quantity dispensed at the refill date of the medication (prescribed daily dosage)		
Refill date		Metformin 850 mg (1–0–1)	Fosinopril 20 mg (1–0–0)	Glibornurid 25 mg (½–0–½)
18.01.11		100*	98	–
18.03.11	59	100*	98	100
14.05.11	57	100	0	0*
27.06.11	44	100	98	100
12.08.11	46	100	0	0
01.10.11	50	100	98	100
21.11.11	51	100*	0	0*
23.01.12	63	100	98	100
01.03.12	38	100	0	0
16.04.12	46	100	98	100
19.06.12	64	100	0	0
02.08.12	44	200*	98	100*
20.11.12	110	100	98	100
31.12.12	41	100	0	0
19.02.13	50	100	98	100
08.04.13	48	100	0	0
25.05.13	47	100	98	100
16.07.13	52	200	0	100
31.07.13	15			
Total	925	1,000	980	1,000
Total gaps		−22	0	−13

Calculation: “average MPR” = [(500 + 980 + 1,000)/3]/925 = 89 %

DPPR = [(50 × 2/2 + 9 × 1/2) + (50 × 3/3 + 7 × 2/3) + (43 × 3/3 + 1 × 2/3) + 46 × 3/3 + 50 × 3/3 + 51 × 3/3 + (53 × 3/3 + 6 × 2/3 + 4 × 1/3) + 38 × 3/3 + 46 × 3/3 + 64 × 3/3 + 44 × 3/3 + (108 × 3/3 + 2 × 1/3) + 41 × 3/3 + 50 × 3/3 + 48 × 3/3 + 47 × 3/3 + 52 × 3/3 + 15 × 3/3]/925 = 99 %

Gaps: mean −1 %

**Table:** 

Patient F2	No days in the interval	Quantity dispensed at the refill date of the medication (prescribed daily dosage)					
Refill date		Gliclazid 30 mg (2–0–0)	Sitagliptin 100 mg (1–0–0)	Aspirin 100 mg (1–0–0)	Valsartan 80 mg (1–0–0)	Simvastatin 80 mg (0–0–½)	Gingko-biloba extract (1–0–1)
04.01.11		120	98	–	–	–	–
10.02.11	37	120	196	–	98	100	240
03.05.11	82	0*	0	28*	0*	0	0*
21.06.11	49	120	0	0*	98	0	120
04.08.11	44	240	0	98	0*	0*	0*
18.10.11	75	0	0*	0	196	0*	0*
08.11.11	21	0	98	0*	0	0*	0*
03.12.11	25	0*	0	0*	0	100	120
03.01.12	31	120*	0*	98	0	0	0*
15.03.12	72	120	98	0*	98	0	0*
14.05.12	60	120	0	0*	0	0	0*
04.06.12	21	0	0*	98	0	0*	0*
25.06.12	21	0*	98	0	98	0*	0*
25.08.12	61	120	98	98	0	100	0*
20.10.12	56	240	0*	98	98	0	240
25.01.13	97	120	98	0	0*	0	0*
25.02.13	31	0	0	0*	0*	0*	120
05.04.13	39	120	98	98	0*	0*	0*
13.05.13	38	0*	0	0*	0*	100	0*
13.07.13	61	0*	98	98	98	0	120
31.07.13	18						
Total	939	780	980	714	784	800	480
Total gaps		−159	−66	−186	−204	−223	−497

Calculation: “average MPR” = [(780 + 980 + 714 + 784 + 800 + 480)/6]/939 = 80 %

DPPR = [37 × 2/2 + 82 × 5/5 + (1 × 6/6 + 15 × 5/6 + 12 × 4/6 + 10 × 3/6 + 11 × 2/6) + 44 × 5/6 + (16 × 6/6 + 9 × 5/6 + 29 × 4/6 + 21 × 3/6) + (7 × 4/6 + 14 × 3/6) + (2 × 4/6 + 23 × 3/6) + (15 × 5/6 + 16 × 4/6) + (29 × 6/6 + 13 × 5/6 + 18 × 4/6 + 12 × 3/6) + (26 × 5/6 + 34 × 4/6) + 21 × 4/6 + (16 × 5/6 + 1 × 4/6 + 4 × 3/6) + (18 × 4/6 + 43 × 3/6) + 56 × 5/6 + (79 × 6/6 + 18 × 5/6) + (23 × 6/6 + 2 × 5/6 + 6 × 4/6) + (16 × 5/6 + 12 × 4/6 + 11 × 3/6 + (13 × 4/6 + 25 × 3/6) + (39 × 4/6 + 21 × 3/6 + 1 × 2/6) + 18 × 5/6)]/939 = 75 %

Gaps: mean −24 %

The results are presented in Box 1. The MPRs were calculated with the average MPR method and yielded 90 % for patient M1, 89 % for patient F1, and 80 % for patient F2. With the DPPR method and the standards defined above, the DDPR rates were 88, 99 and 75 %, respectively. The mean numbers of days without supply (gaps) were −10, −1 and −24 %, respectively.

## Discussion

The two methods most often used to measure medication adherence from dispensing data records are the MPR and the PDC. However, because of lack of standards and definitions necessary for the parameters used in calculation, the methods described in studies vary widely. As an example, Hess et al. [18] calculated adherence rates ranging from 63.5 to 104.8 % when applying 11 different calculation methods to the same set of pharmacy data. As a consequence, comparing results between studies is often difficult if not impossible. Further, many assumptions are made when adherence rates (i.e., medication consumption) are calculated from secondary databases; e.g., that a person has the medication available on the day of the prescription; that patients consume the medication as prescribed; that patients start taking the medication on the day of dispensation until the supply is exhausted; that medication consumption is consistent throughout the observation period; or that all extra doses accumulated during the observation period are taken by the patient until depleted if refills are not obtained on time or before the next refill [19]. This might explain why some studies specify corrections for values, often without a clear rationale, like setting negative values to zero [17] or multiplying duration of drug use by factor 1.1 to control for irregular use and early drug dispensation [20].

To enable a more uniform presentation of data and thus improve the consistency and quality of adherence analyses, international experts developed a checklist of key issues on how to perform retrospective analyses of refill medication databases [21]. Unfortunately, the proposed measurements of adherence lack key details and procedures, such as rules to avoid double-counting covered days or handling oversupply. It is evident that accurate calculation of adherence rates from refill data requires standard definitions of the considered time frame, the numerator and denominator, and the management of missing values and/or time periods. A more subtle calculation has been advocated [16] to allow for the comparison of results across studies and the translation to real world practice. With the advance of computerized pharmacy records, some researchers developed computational frameworks to detect such events as medication lapses in refill databases [22]. However, these technical developments are only useful for individual patient information and need further evaluation.

The influence of variable terms on the assessment of adherence to single drug is amplified with multiple drug assessment, especially when the method used is indifferent to the specific settings. A study comparing different calculation methods showed that the use of MPR for more than one medication overestimates adherence, predominantly due to the presence of duplication [4]. Since the “average MPR” does not account for the number of medications, the frequency of medication switching, the duplication, the overlapping, or the unexpected and same-day refills, it can hardly reflect the actual adherence that it was intended to measure. Thus, MPR methods are inadequate for quantifying adherence to polypharmacy regimens.

In this article, we defined new standards for the calculation of possession rates with refill data and proposed a new index, the DPPR. This index considers the presence or absence of multiple medications on each day in the observation period. It quantifies polypharmacy adherence as the percentage of medications daily available. This approach accounts for the specificity of polypharmacy such as the number of medications and frequency of medication switching. It also eliminates duplication and overlapping, the parameters responsible for the general overestimation of adherence. With the three illustrative cases we selected in a community pharmacy over 31 months, we piloted the new method and demonstrated its face validity in daily practice. As predicted, the DPPR values were lower than the average MPR estimates. Thus, we posit that the DPPR is closer to the actual adherence rate than other calculations.

Our approach has several strengths. First, we propose a standardization of the parameters used for calculation. Second, we propose a method that is insensitive to oversupply and duplication, the two parameters in mathematical calculations that lead to overestimation of adherence rates. Third, the DPPR represents a continuous index of adherence across all subjects rather than a threshold-based index, separating adherent from non-adherent subjects. Moreover, the conversion from continuous data into categorical data as well as the use of cut-points is only recommended when the clinical validity of the specified level of adherence has been demonstrated [21]. To our knowledge, this exists only for oral contraceptives and HIV drugs [23].

Our new index also has limits. First, the DPPR cannot detect oversupply. Thus, we propose to indicate additionally the evaluation of the accumulated surplus (oversupply) and the accumulated days with at least one missing medication (gaps). Second, the DPPR requires a “supply diary” for each patient-day. This may be difficult to generate by computer, mainly because dispensing and recording services may differ across countries. For example, European pharmacies dispense manufactured packagings of varying sizes while US pharmacies have access to bulks and dispense the exact number of units prescribed. The maximum quantity of dispensed drugs is usually 90 days in the Netherlands, with a maximum of 15 days for the first dispensing, while no such restriction exists in Switzerland or Germany, where the first dispensed package can be 100 tablets in size. Finally, calculation with variable dosage schedule (e.g., “take 1 or 2 pills…”) or “as needed” as part of the instructions is not possible and these medications are to be disregarded from the evaluation.

## Conclusion

Estimates of adherence to single-medications obtained from MPR-based methods may vary because of differences in calculation methods. This problem is amplified by multiple factors outlined in this article. Because adherence to multiple medications has been assessed with methods developed for single-medication use, results have so far proved divergent. We propose new definitions to standardize the parameters needed to calculate possession rates with secondary databases. We further propose a new method to calculate possession rate with multiple medications that accounts for the specificity of polypharmacy. Studies are needed to validate the new index DPPR, preferably with a national database. Subsequently, defining of a formula and programming of codes for computer-generating the DPPR from dispensing data records should be considered.
